# PGP-5 Influences ZIP-2-Dependent Innate Immune Response During *Streptococcus gordonii* Exposure in *Caenorhabditis elegans*

**DOI:** 10.3390/cimb48101059

**Published:** 2026-10-14

**Authors:** Boopathi Balasubramaniam, Judith Leon Jaramillo, Julia Stefanik, Thy Thy Nguyen, Mandi Morris, Ransome van der Hoeven

**Affiliations:** 1 Iowa Institute for Oral Health Research, University of Iowa College of Dentistry, Iowa City, IA 52242, USA; 2 Katz Department of Oral and Maxillofacial Surgery, School of Dentistry, University of Texas Health Science Center, Houston, TX 77054, USA; 3 Department of Periodontics, University of Iowa College of Dentistry, Iowa City, IA 52242, USA

**Keywords:** *C. elegans*, *pgp-5*, *zip-2*, *irg-1*, innate immunity

## Abstract

Innate immune responses in *Caenorhabditis elegans* are regulated by multiple effectors that are activated upon bacterial exposure. In this study, we investigated the role of *pgp-5*, a transmembrane efflux transporter implicated in cellular stress responses and detoxification in host defense against *Streptococcus gordonii*. *S. gordonii* produces hydrogen peroxide (H_2_O_2_) as a major virulence factor and induces oxidative stress in host cells. Exposure to wild-type *S. gordonii* induced robust *pgp-5p*::GFP expression, whereas the H_2_O_2_-deficient *spxB* mutant elicited significantly lower reporter activity, suggesting that *pgp-5* activation is associated with *spxB*-dependent H_2_O_2_ production. Previous studies have established that the bZIP transcription factor ZIP-2 is required for *pgp-5* expression in *C. elegans* under xenobiotic stress conditions. However, whether pathogen-induced *pgp-5* expression similarly depends on ZIP-2 remains unknown. In addition, the potential role of *pgp-5* in regulating ZIP-2-dependent immune responses has not been explored. To address these questions, we examined *pgp-5p*::GFP expression and activation of the ZIP-2-dependent innate immune reporter *irg-1p*::GFP following RNAi-mediated knockdown of *pgp-5* and *zip-2* during exposure to wild-type and *spxB* mutant *S. gordonii*. Knockdown of *zip-2* abolished *S. gordonii*-induced *pgp-5p*::GFP expression compared with empty vector control-treated worms, demonstrating that pathogen-induced *pgp-5* expression is dependent on ZIP-2. In contrast, knockdown of *pgp-5* resulted in significantly increased expression of *irg-1p*::GFP following *S. gordonii* exposure. These findings suggest that loss of PGP-5 enhances expression of the ZIP-2-responsive gene *irg-1* under *S. gordonii*-induced stress, indicating that PGP-5 may influence the magnitude of the associated immune response in *C. elegans*.

## 1. Introduction

Living organisms face constant threats from pathogenic microbes, and their ability to survive infection depends on well-coordinated immune defenses and mechanisms of detoxifying pathogen-derived toxins and virulence-associated stresses. Over the past several decades, the soil nematode *C. elegans* has been used as an outstanding host model to study innate immunity against various bacterial infections [1,2]. Upon pathogen exposure, *C. elegans* activates a diverse repertoire of immune effectors through multiple signaling pathways, including the highly conserved p38-MAPK pathway [3,4,5]. Notably, different pathogens elicit distinct host responses, highlighting the context-dependent specificity of the worm immune system and adaptation to microbial threats. Among various bacterial pathogen models, members of the genus *Streptococcus* have been particularly valuable for investigating oxidative stress-mediated host responses largely because they produce H_2_O_2_ as a major virulence factor through the activity of *spxB*, a pyruvate oxidase gene [6,7,8]. *S. gordonii,* a member of mitis-group streptococci, is a common constituent of the human oral microbiota but can become pathogenic upon entering the bloodstream [9]. Clinically, *S. gordonii* has been implicated in several human diseases, including infective endocarditis, orbital cellulitis, and bacteremia, particularly in immunocompromised or neutropenic patients [10]. Furthermore, invasive viridans group streptococcal infections have become increasingly recognized in neutropenic patients, with antimicrobial resistance contributing to adverse clinical outcomes [11,12]. Despite its clinical importance, the molecular mechanisms underlying *S. gordonii* pathogenesis and host–pathogen interactions remain incompletely understood. We previously demonstrated that H_2_O_2_ production directly induces oxidative stress in *C. elegans* and activates SKN-1 through the p38-MAPK signaling pathway [6]. However, our understanding of additional host signaling pathways and downstream effectors involved in the response to *S. gordonii* infection remains limited. Little is known how host protective mechanisms beyond canonical stress-response pathways contribute to adaptation and survival during oxidative stress-inducing infections. In this context, one underexplored aspect of host immunity is the role of transmembrane efflux transporters in protecting cells from pathogen-induced cellular stress. Members of the ABC transporter family, particularly P-glycoproteins, function as efflux pumps exporting toxic compounds out of cells which are critical during both xenobiotic detoxification and pathogenic stress adaptation [13,14,15,16,17]. In *C. elegans*, *pgp-5* encodes a P-glycoprotein family transporter that is strongly induced by bacterial infection and diverse xenobiotic stress, including heavy metal exposure [18,19,20,21,22]. Related P-glycoprotein family genes, including *pgp-3*, *pgp-4*, *pgp-6* and *pgp-7*, are also regulated under xenobiotic stress and bacterial exposure, including Ivermectin-resistant strains [23,24]. However, *pgp-5* is the most extensively characterized xenobiotic-responsive ABC transporter and exhibits robust induction across multiple stress conditions, emerging as a widely used reporter of xenobiotic stress and detoxification responses [16,18,19,20,21].

Among the different innate immune pathways in *C. elegans*, the bZIP transcription factor ZIP-2 represents a distinct pathogen-responsive immune pathway that has been extensively characterized during *Pseudomonas aeruginosa* infection [3,25,26]. Unlike the classical p38-MAPK-mediated immune pathway, ZIP-2 activation occurs through a unique mechanism involving pathogen-induced cellular stress, particularly inhibition of host translation. Under basal conditions, ZIP-2 protein is unstable and undergoes degradation in the cytoplasm [27]. However, during bacterial infection, ZIP-2 becomes stabilized, allowing its accumulation and translocation into the nucleus, where it promotes the expression of infection-responsive genes, including *irg-1* [26]. Although initially identified as a key regulator of host defense during *P. aeruginosa* infection, subsequent studies have shown that ZIP-2 also contributes to cellular stress responses, including responses to xenobiotic and proteotoxic stress, highlighting its broader role in cellular stress adaptation [21,28]. Emerging evidence suggests a functional relationship among ZIP-2, p38-MAPK, and PGP-5 are closely associated in coordinating innate immune responses and detoxification processes in *C. elegans*. Mechanistically, it has been shown that PGP-5 induction is regulated by p38-MAPK signaling under stress conditions. In parallel, ZIP-2 is also required for the PGP-5 expression under stress conditions [19,21]. However, ZIP-2-mediated induction of *pgp-5* occurs independently of the p38-MAPK pathway, with both pathways functioning as separate regulators of stress-responsive gene expression. Consistent with a protective role during infection, loss of *pgp-5* has been associated with increased susceptibility to bacterial pathogens. However, the mechanisms underlying this phenotype remain poorly understood [19,20]. It is unclear whether PGP-5 promotes host survival primarily through its predicted function as a transmembrane efflux transporter that removes toxic compounds, or whether it additionally influences stress signaling and immune regulation. In this study, we address this using *S. gordonii* wild-type (WT) and the *spxB* mutant strain that is deficient in H_2_O_2_ production. Because *S. gordonii*-derived H_2_O_2_ is a major source of pathogen-induced oxidative stress, we hypothesized that PGP-5 contributes to host protection by mitigating the effects of oxidative stress and associated toxic byproducts generated during infection. We therefore investigated how *pgp-5* affects host survival and expression of the *zip-2*-dependent innate immune reporter *irg-1* in the presence of *S. gordonii*. Our findings indicate that PGP-5 functions not only as a stress-responsive transporter but also as a modulator of immune signaling. Specifically, loss of *pgp-5* enhances *irg-1* induction, suggesting that PGP-5 contributes to host stress tolerance and may influence stress-responsive immune signaling associated with survival during *S. gordonii* exposure.

## 2. Materials and Methods

### 2.1. Worm Strains and Bacterial Strains

The *C. elegans* strains used in this study were acquired from the Caenorhabditis Genetics Center (CGC). Worm strains were maintained and propagated on nematode growth medium (NGM) under standard laboratory conditions at 20 °C [29]. The strains included N2 Bristol; RB959—*pgp-5*(*ok856*) (3X backcrossed); VC3056—*zip-2*(*ok3730*) (4X backcrossed); WE5172—*ajIs1* [*rCesC05A9.1*::GFP + *dpy-5*(+)]; AU133—*agIs17* [*myo-2p*::mCherry + *irg-1p::GFP*]. For age synchronization, gravid adults were harvested from well-grown plates and subjected to alkaline hypochlorite treatment (sodium hypochlorite mixed with sodium hydroxide in a 4:1 ratio) to release embryos. The collected embryos were centrifuged, and the resulting pellets were washed 3 times with M9 buffer and incubated in M9 overnight to allow synchronous hatching. The resulting L1 larvae were transferred to fresh plates and grown at 20 °C until they reached the L4 developmental stage for subsequent experiments.

*S. gordonii* strains were grown in Todd Hewitt broth supplemented with 0.3% yeast extract (THY), while *Escherichia coli* strains were maintained in Luria–Bertani (LB) medium with applicable antibiotics, including streptomycin or carbenicillin. The *S. gordonii* strains used included the wild-type DL1 Challis, the *spxB* deletion mutant (DL1 Δ*spxB*), and the WT copy of the *spxB* complemented strain (Δ*spxB*;*spxB^+^*) [6,30].

### 2.2. Survival Assay and Catalase Treatment

Overnight *Streptococcus* cultures were prepared in THY medium for 16 h and seeded onto THY agar medium [6,30]. For catalase treatment, 1000 U of catalase was dissolved in sterile water, filter-sterilized, and spread onto THY agar plates. The plates were allowed to dry before WT *S. gordonii* was seeded. All pathogen-seeded plates were incubated for 18 h at 37 °C in a candle jar. Synchronized L4 larvae from three independent populations were transferred onto the respective bacterial lawns (20 worms per plate; three independent replicate plates per condition). Worms were then incubated at 22 °C, and survival rate was scored at defined intervals during the assay. Worms that failed to react to gentle touch with a sterile worm pick were scored dead. Survival data were analyzed using Kaplan–Meier analysis based on the survival times of individual worms, with differences between groups assessed using the log-rank (Mantel–Cox) test. Each condition included 20 worms per plate across three replicate plates (60 worms total).

### 2.3. Fluorescence Microscopy and Image Analysis

To assess transgene expression, at least 100 worms were transferred to the indicated bacterial strains as mentioned above and incubated for 2.5 h at 22 °C. Worms were then washed twice with sterile M9 buffer and anesthetized with 2 mM sodium azide. Anesthetized worms were placed on 5% agarose pads covered with microscope coverslips, and fluorescence imaging was performed using an Olympus BX-63 upright microscope (Olympus, Tokyo, Japan) [6,31]. All images were collected under consistent light intensity and exposure parameters for all experimental conditions. Fluorescence mean intensity levels were quantified using ImageJ software (Version 1.54k). Subtraction of background fluorescence was done using the default rolling ball radius (50 pixels). A polygon selection was drawn around the intestinal region while excluding the pharynx, and the mean fluorescence intensity within the selected region was measured and plotted.

### 2.4. RNA Interference (RNAi)

RNAi-mediated gene knockdown was performed following established protocols. Briefly, synchronized L1-stage worms were fed *E. coli* HT115(DE3) bacteria expressing target-specific dsRNA and maintained until the L4/young adult stage [29,32]. RNAi bacteria were cultured overnight in LB medium containing carbenicillin at 37 °C with gentle agitation. The bacterial cultures were then seeded onto RNAi induction plates (IPTG + carbenicillin) and incubated at 37 °C overnight before use. Synchronized L1 worms were transferred onto the RNAi induction plates and maintained until the L4/young adult stage. Subsequently, RNAi-treated worms were washed from the RNAi plates using sterile M9 buffer, transferred to pathogen exposure plates as described above, and exposed for 2.5 h before fluorescence imaging. RNAi clones were obtained from the *C. elegans* RNAi library (Source BioScience, UK Limited, Nottingham, Nottinghamshire, UK).

### 2.5. RNA Extraction, cDNA Synthesis, and Quantitative Reverse Transcription-PCR (qRT-PCR)

Synchronized L4-stage worms were transferred to the specific bacterial lawns and incubated for 2.5 h at 22 °C. Following experimental exposures, RNA samples were prepared by extracting total RNA with TRIzol™ reagent (Invitrogen™, Carlsbad, CA, USA) according to the manufacturer’s recommended protocol. RNA concentration and purity were determined prior to downstream analysis using Thermo Scientific™ NanoDrop™ One/One^C^ Microvolume UV-Vis Spectrophotometer (Thermo Fisher Scientific, Madison, WI, USA). RNA samples were normalized to equal concentrations and subjected to DNase treatment. First-strand cDNA was then synthesized using the Maxima First Strand cDNA Synthesis Kit with dsDNase (Thermo Fisher Scientific Baltics UAB, Vilnius, Lithuania) following the manufacturer’s instructions. To analyze gene expression levels, qRT-PCR was performed using the iTaq Universal SYBR Green Supermix (Bio-Rad Laboratories, Inc., Hercules, CA, USA) on a Bio-Rad CFX Opus 96 Touch Real-Time PCR Detection System (Bio-Rad laboratories, Singapore). Relative gene expression levels were calculated using the comparative Ct (2^−ΔΔCt^) method, with *act-1* serving as the internal reference gene for normalization [29]. Primer details are given in Table 1. Gene expression in pathogenic bacteria-exposed worms was normalized to the corresponding control group. Each experiment was performed with three independent biological replicates.

### 2.6. Statistical Analysis

All statistical analyses were performed using GraphPad Prism version 11.0 (GraphPad Software). Survival data are presented as mean ± SEM. Survival curves were analyzed using Kaplan–Meier analysis with the log-rank (Mantel–Cox) test. One-way or two-way ANOVA followed by appropriate multiple comparisons tests or unpaired two-tailed *t*-tests were used where indicated. The specific statistical test used for each experiment is described in the corresponding figure legends.

## 3. Results

### 3.1. Loss of pgp-5 Is Associated with Increased Sensitivity to WT S. gordonii Exposure

**Figure 1:**
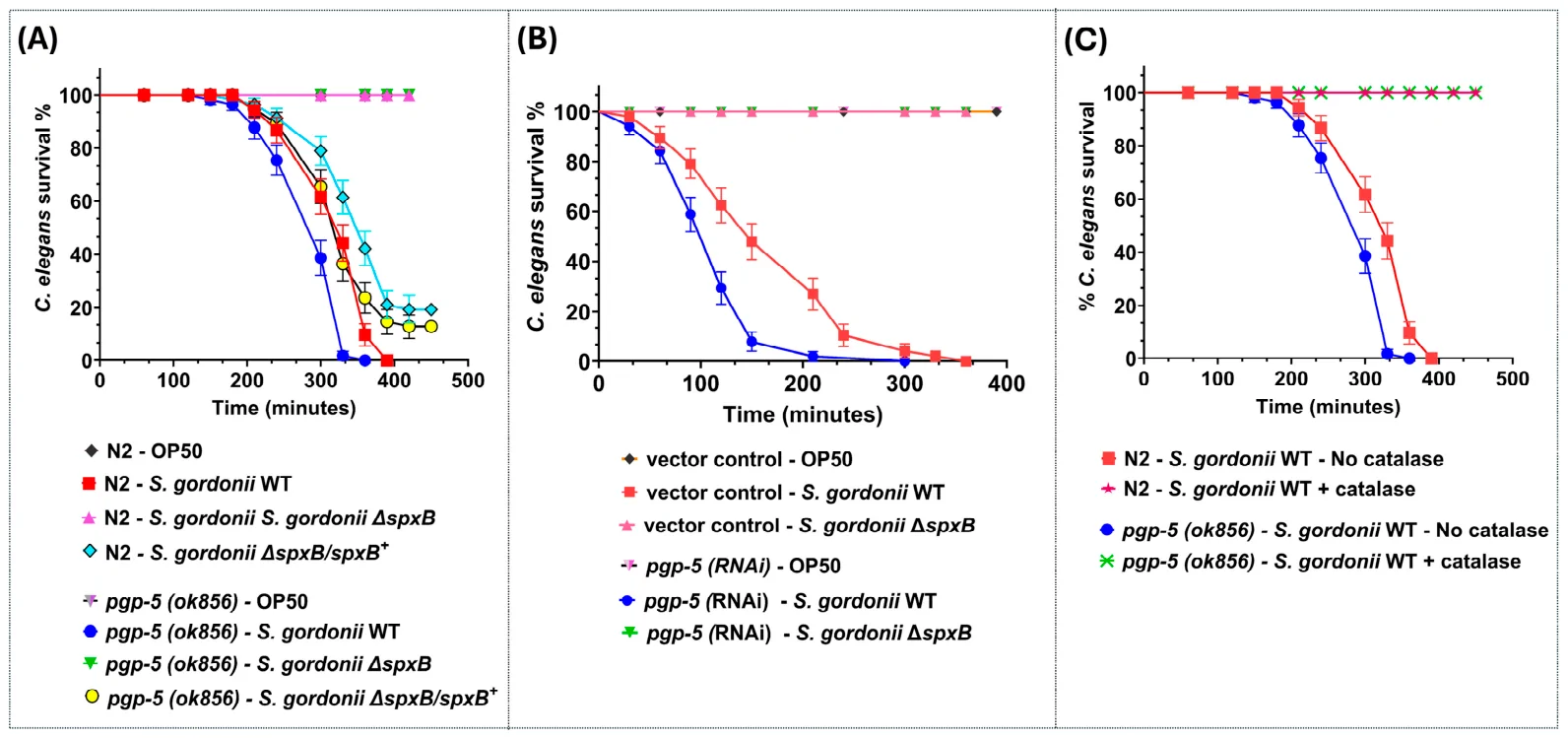
(**A**) Survival percentage of N2 and *pgp-5(ok856)* worms following exposure to WT *S. gordonii*, Δ*spxB* mutant or *spxB* complemented strain (*p* < 0.05, *n* = 60 worms). (**B**) Survival percentage of empty vector RNAi control or RNAi-mediated knockdown of *pgp-5* worms exposed to WT *S. gordonii* and H_2_O_2_-deficient mutant or Δ*spxB* strain (*p* < 0.05, *n* = 60 worms). (**C**) Survival percentage of N2 and *pgp-5*(*ok856*) worms following exposure to WT *S. gordonii* in the presence or absence of catalase (*p* < 0.05, *n* = 60 worms). The survival data were analyzed by Kaplan–Meier survival analysis, and variations between groups were assessed using the log-rank (Mantel–Cox) statistical test.

We first examined whether *pgp-5* contributes to *C. elegans* survival during *S. gordonii* exposure. *pgp-5*(*ok856*) and N2 worms were exposed to WT *S. gordonii*, the *spxB* mutant strain and the *spxB-*complemented strain to determine the contribution of bacterial H_2_O_2_ production to host susceptibility. Upon exposure to WT *S. gordonii*, *pgp-5*(*ok856*) mutant showed an increased sensitivity and reduced survival compared with N2 worms (Figure 1A). However, no noticeable killing was observed in both worm strains following exposure to the *spxB* mutant strain. We noticed a significant decline in the survival rate following the exposure to the *spxB-*complemented strain in both worm strains. We observed a similar trend with RNAi-mediated knockdown of *pgp-5* as well (Figure 1B). We next used catalase treatment to assess the contribution of bacterial H_2_O_2_ produced by WT *S. gordonii* to host survival. Catalase treatment significantly improved worm survival compared with WT *S. gordonii* exposure (Figure 1C), supporting a role for bacterial H_2_O_2_ in the observed phenotype. Altogether, these results indicate that *pgp-5* contributes to host protection during *S. gordonii* exposure and is important for tolerance to H_2_O_2_-associated bacterial stress.

### 3.2. Bacterial H_2_O_2_ Is Involved in S. gordonii-Induced pgp-5p::GFP Expression

**Figure 2:**
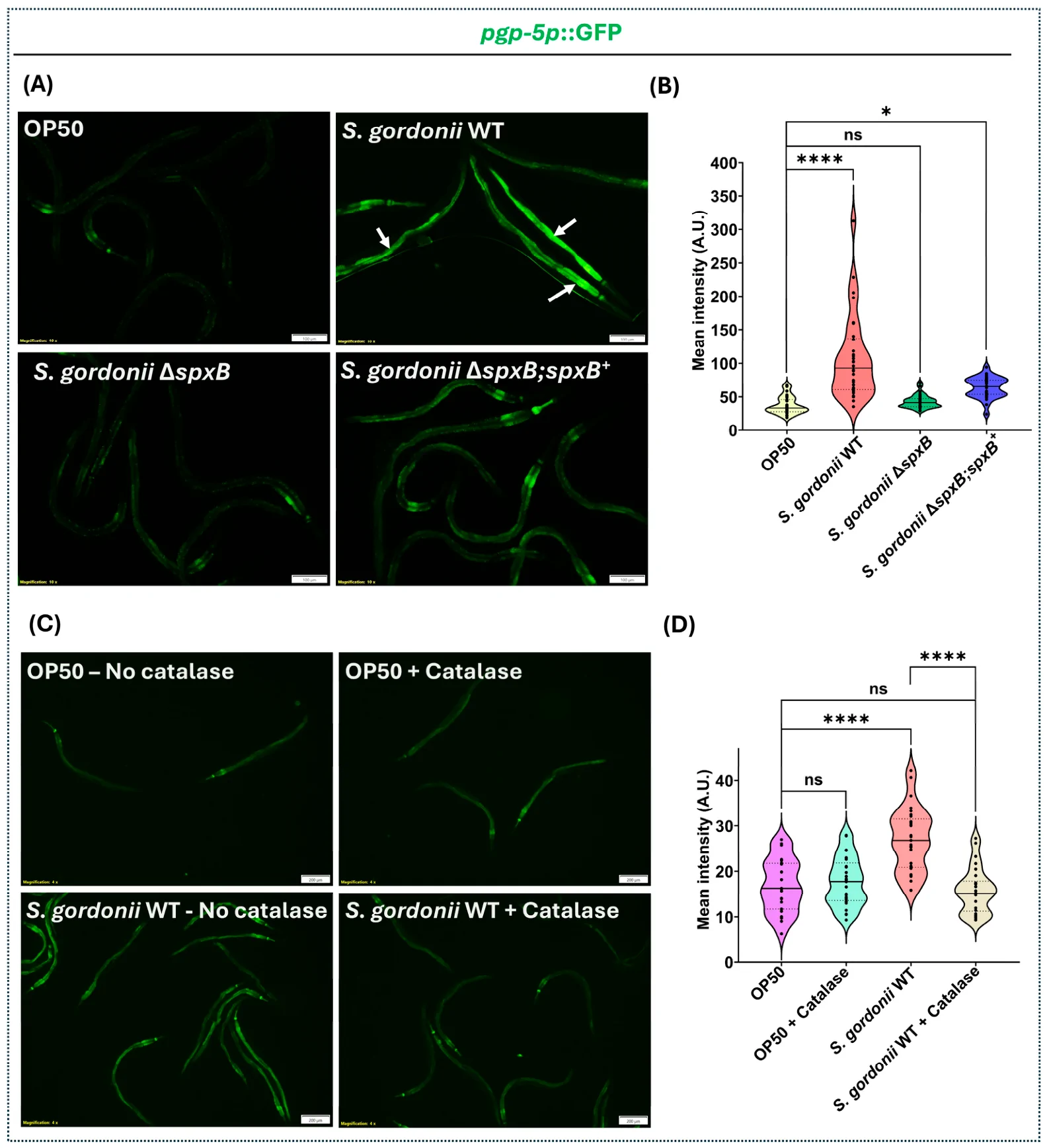
(**A**) Representative microscopic images showing *pgp-5p::*GFP expression following *S. gordonii* exposures. Scale bar = 100 µm, magnification = 10×. Arrows indicate the induced GFP expression pattern. (**B**) Quantification of mean fluorescence intensity indicating H_2_O_2_-mediated induction of *pgp-5p::*GFP expression following *S. gordonii* exposures (*n* ≥ 25). (**C**) Representative microscopic images showing *pgp-5p::*GFP expression following *S. gordonii* exposure in the presence or absence of catalase. Scale bar = 200 µm, magnification = 4×. (**D**) Quantification of mean fluorescence intensity showing significant reduction in *S. gordonii*-induced *pgp-5p::*GFP expression following catalase treatment (*n* ≥ 20). Each dot represents one worm. Violin plots depict the distribution of fluorescence intensity values, with the center bold line indicating the median. Statistical significance was assessed using one-way ANOVA followed by Dunnett’s multiple comparisons test, with each bacterial condition compared against the OP50 control. * *p* < 0.05, **** *p* < 0.0001; ns—not significant.

To determine whether *pgp-5* expression is induced upon *S. gordonii* exposure and whether this response depends on bacterial H_2_O_2_ production, we analyzed *pgp-5p::*GFP transcriptional reporter expression using fluorescence microscopy. Under OP50 conditions, worms showed basal levels of *pgp-5p*::GFP expression. Exposure to WT *S. gordonii* resulted in a significant increase in GFP expression compared with OP50 controls. In contrast, worms exposed to the *S. gordonii spxB* mutant showed no significant difference compared with OP50 and displayed significantly reduced GFP expression compared with the WT *S. gordonii*. Complementation of *spxB* restored the induction of *pgp-5p::GFP* expression, with levels significantly higher than those observed in OP50 and the *spxB* mutant conditions (Figure 2A,B). We next treated the worms with catalase to test whether bacterial H_2_O_2_ contributes to *pgp-5p::GFP* expression. As shown in Figure 2C,D, catalase treatment significantly reduced *pgp-5p::GFP* expression to levels comparable to those observed in OP50-fed worms. These results demonstrate that *pgp-5* expression is specifically induced during *S. gordonii* exposure and is dependent on streptococcal-derived H_2_O_2_.

### 3.3. H_2_O_2_ Contributes to S. gordonii-Induced irg-1p::GFP Expression

**Figure 3:**
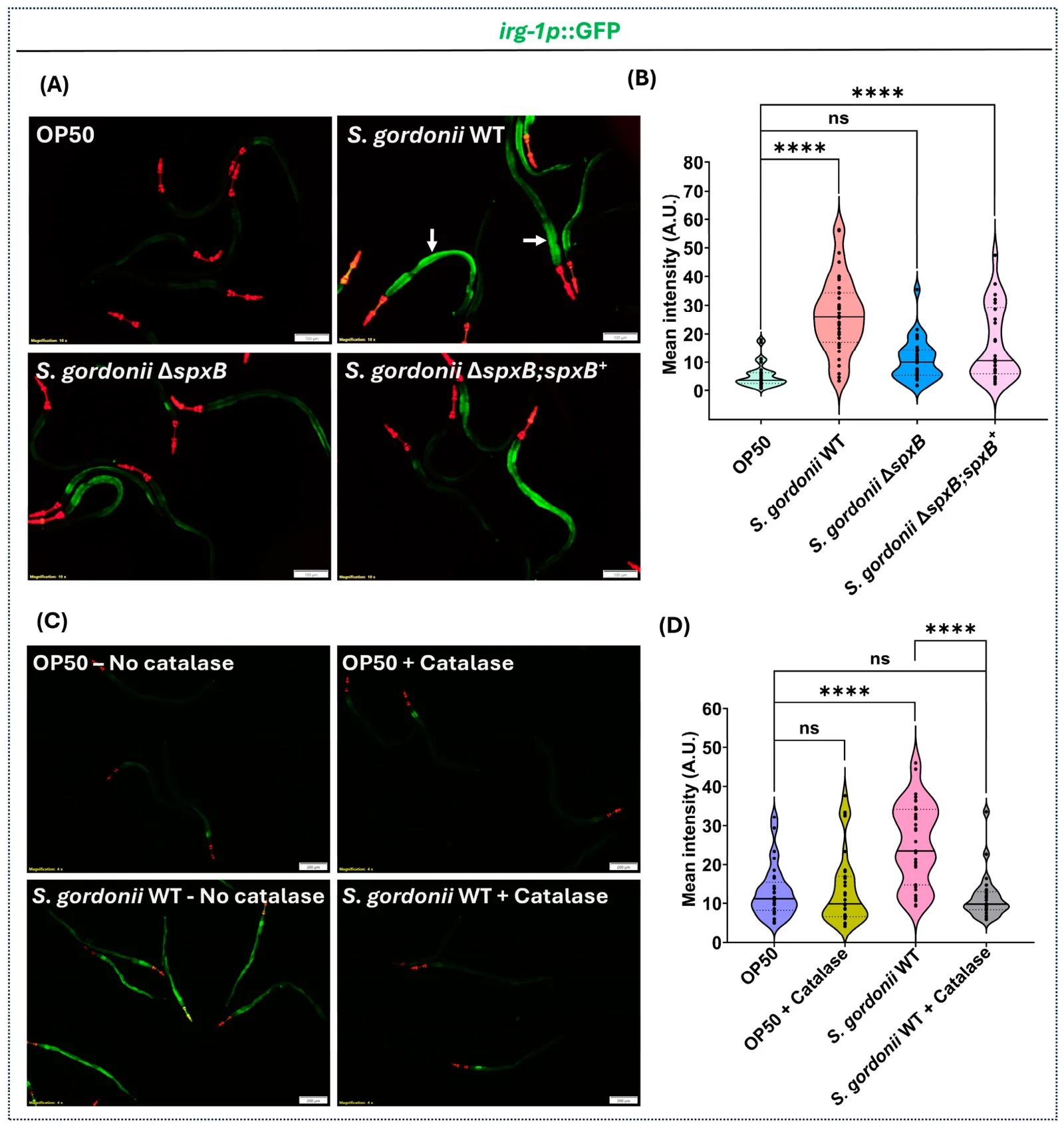
(**A**) Representative microscopic images showing *irg-1p::GFP* expression upon *S. gordonii* exposures. Scale bar = 100 µm, magnification = 10×. Arrows indicate the induced GFP expression pattern. (**B**) Quantification of mean fluorescence intensity demonstrating H_2_O_2_-mediated induction of *irg-1p::GFP* expression following *S. gordonii* exposures. (*n* ≥ 25). (**C**) Representative images of *irg-1p::GFP* expression following *S. gordonii* exposure with or without catalase treatment. Scale bar = 200 µm; magnification = 4×. (**D**) Quantification of mean fluorescence intensity showing reduced *irg-1p::GFP* expression following catalase treatment (*n* ≥ 25). Each dot represents one worm. Violin plots depict the distribution of fluorescence intensity values, with the center bold line indicating the median. Statistical significance was assessed using one-way ANOVA followed by Dunnett’s multiple comparisons test, with each bacterial condition compared against the OP50 control. **** *p* < 0.0001; ns—not significant.

It is well established that ZIP-2 functions as a key transcriptional regulator of innate immune response genes, such as *irg-1*, in *C. elegans* [26]. To determine whether *irg-1* expression is induced during *S. gordonii* exposure and whether this response depends on bacterial H_2_O_2_ production, we analyzed the expression of the *irg-1p::*GFP reporter marker. Under OP50 conditions, worms displayed basal levels of *irg-1p::*GFP expression, whereas WT *S. gordonii* exposure showed a significant increase in GFP fluorescence. *S. gordonii spxB* mutant did not show a significant difference compared to OP50 and exhibited significantly less GFP expression compared with the WT *S. gordonii* strain. *S. gordonii spxB* complemented strain restored *irg-1p::GFP* induction, resulting in increased GFP expression compared with both OP50 and the *spxB* mutant conditions (Figure 3A,B). We next asked whether H_2_O_2_ produced by *S. gordonii* was involved in the induction of the *irg-1* reporter marker. Treatment with catalase significantly decreased *irg-1p::GFP* fluorescence in WT *S. gordonii*-exposed worms, with expression comparable to those of the OP50 control (Figure 3C,D). These data suggest that *irg-1* expression is induced during *S. gordonii* exposure and is dependent on pathogen-derived H_2_O_2_.

### 3.4. Activation of pgp-5 During S. gordonii Exposure Requires the bZIP Transcription Factor zip-2

**Figure 4:**
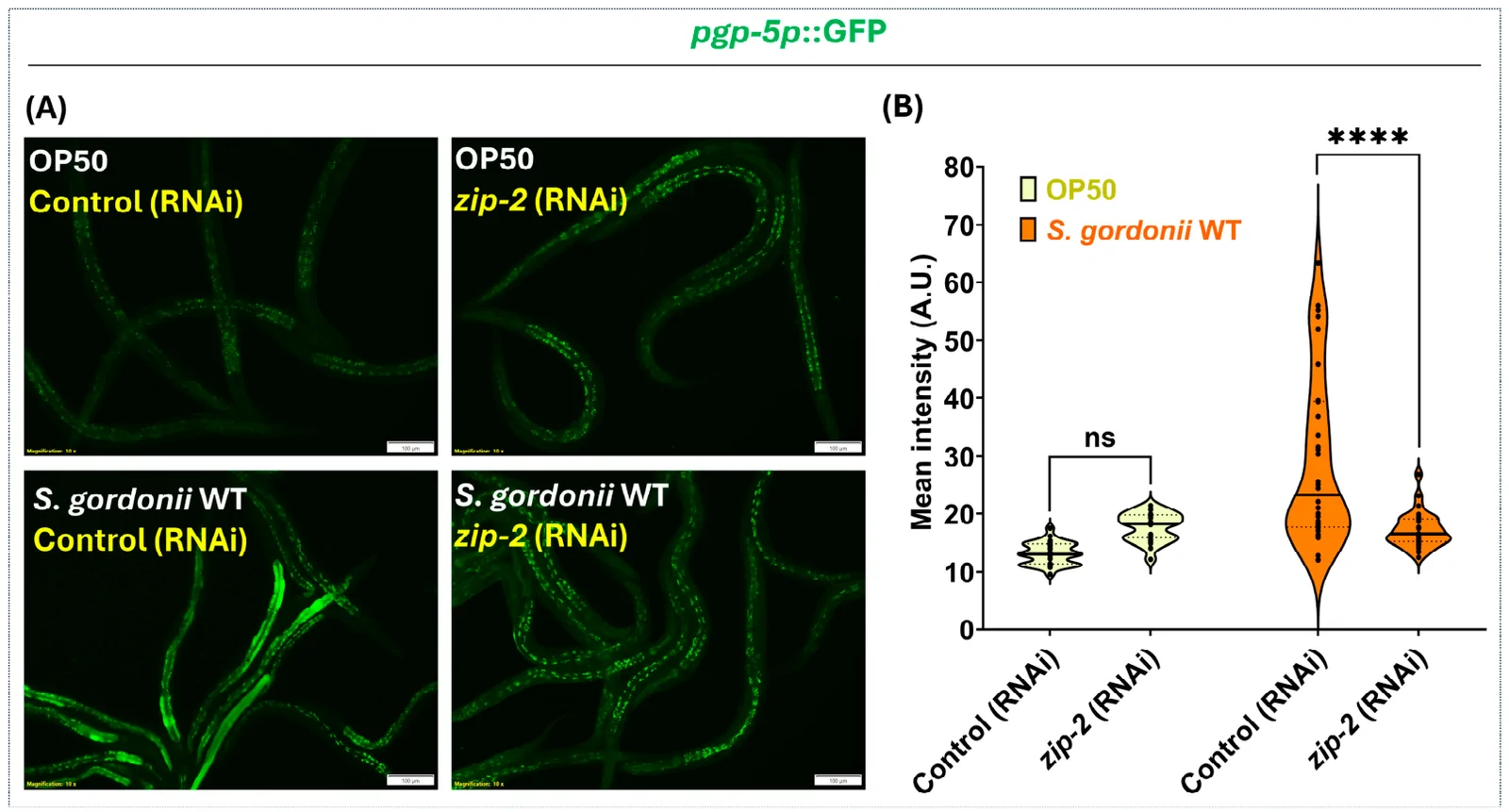
(**A**). Representative microscopic images displaying significantly reduced *pgp-5p::*GFP expression upon *zip-2* RNAi during WT *S. gordonii* exposure. Empty vector RNAi serves as control in this experiment. Scale bar = 100 µm, magnification = 10×. (**B**) Quantification of mean fluorescence intensity showing that *zip-2* is required for *pgp-5p::*GFP induction in response to *S. gordonii* (*n* ≥ 25). Each dot represents one worm. Violin plots depict the distribution of fluorescence intensity values, with the center bold line indicating the median. Statistical significance was determined by two-way ANOVA considering bacterial treatment and RNAi condition as independent factors, followed by Šídák’s multiple comparisons test to evaluate differences among RNAi conditions within each bacterial exposure. **** *p* < 0.0001; ns—not significant.

Previous studies have established that the bZIP transcription factor ZIP-2 is essential for the induction of *pgp-5* under xenobiotic stress conditions [19,21]. In contrast, the regulatory role of ZIP-2 in *pgp-5* expression during bacterial infection/exposure remains unclear. To address this knowledge gap, we tested whether induction of *pgp-5* during *S. gordonii* exposure requires ZIP-2 by examining *pgp-5p*::GFP reporter expression following RNAi-mediated knockdown of *zip-2.* Under OP50 feeding conditions, worms treated with the empty vector RNAi control exhibited only basal *pgp-5p*::GFP expression. In contrast, exposure to WT *S. gordonii* resulted in a significant increase in GFP fluorescence, indicating robust induction of *pgp-5* in response to pathogen exposure. However, *pgp-5p*::GFP fluorescence was markedly reduced in *zip-2* knockdown worms following exposure to *S. gordonii* compared with empty vector controls (Figure 4A,B). Consistent with previous studies showing a role for ZIP-2 in regulating *pgp-5* expression during xenobiotic stress, our results extend this regulatory relationship to bacterial exposure and indicate that ZIP-2 serves as a key upstream regulator of *pgp-5* under both xenobiotic and pathogen-induced stress conditions.

### 3.5. pgp-5 Knockdown Elevates irg-1p::GFP Expression in Response to WT S. gordonii

**Figure 5:**
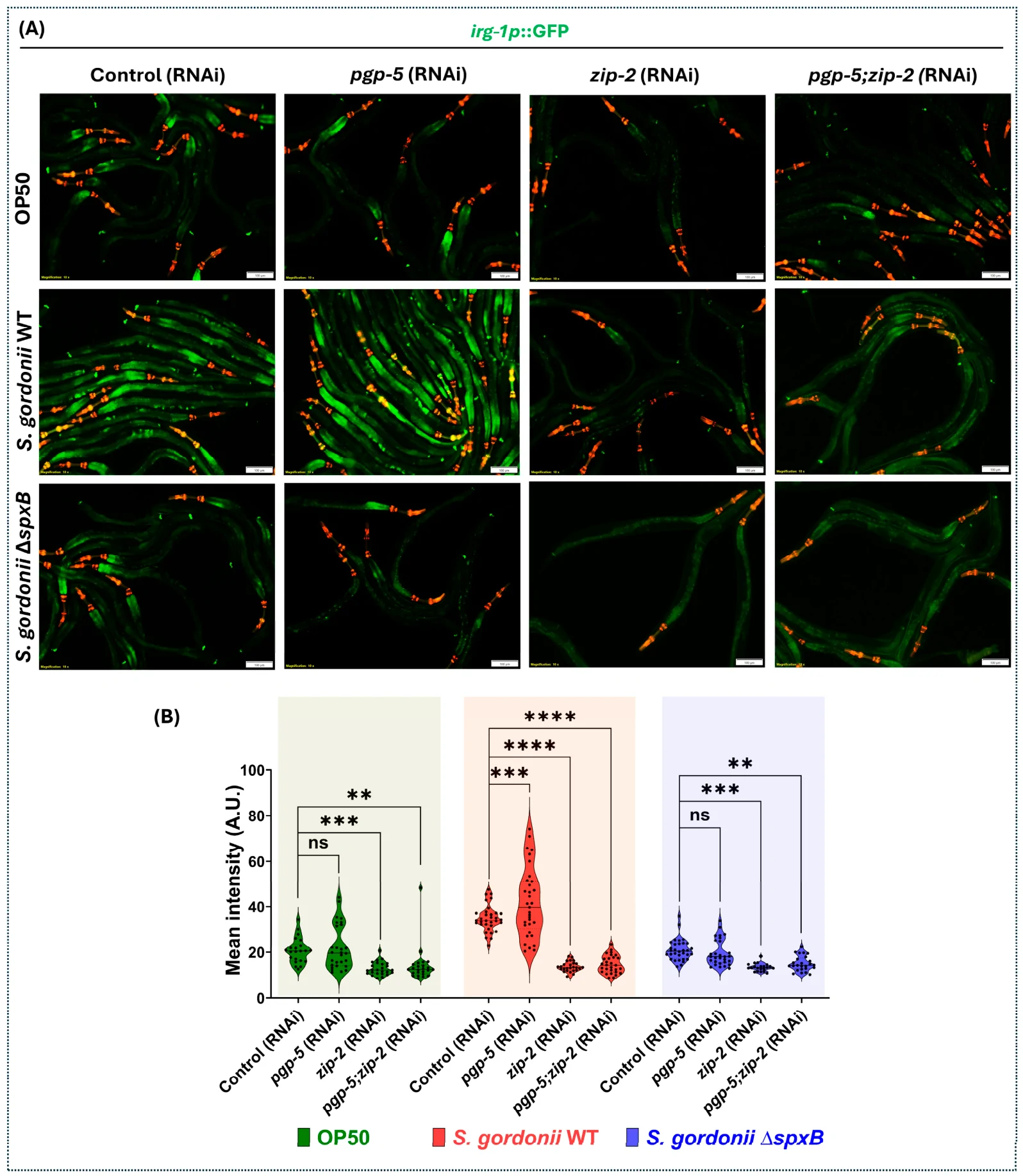
(**A**) Representative microscopic images showing significantly increased *irg-1p::*GFP expression upon *pgp-5* RNAi during WT *S. gordonii* exposure compared with vector control. *zip-2* RNAi acts as a negative control. Scale bar = 100 µm, magnification = 10×. (**B**) Quantification of mean fluorescence intensity confirming significantly enhanced *irg-1p::*GFP induction following *pgp-5* knockdown upon WT *S. gordonii* exposure (*n* ≥ 25). Each dot represents one worm. Violin plots depict the distribution of fluorescence intensity values, with the center line indicating the median. Statistical significance was determined by two-way ANOVA considering bacterial treatment and RNAi condition as independent factors, followed by Tukey’s multiple comparisons test to evaluate differences among RNAi conditions within each bacterial treatment. ** *p* < 0.01, *** *p* < 0.001, **** *p* < 0.0001; ns—not significant.

Our results demonstrated that ZIP-2 is required for the induction of *pgp-5* during *S. gordonii* exposure (Figure 4). However, whether *pgp-5* itself influences ZIP-2-dependent immune activation remains unknown. To better understand this question, we examined the effect of *pgp-5* knockdown on the expression of ZIP-2-dependent immune reporter *irg-1p*::GFP following WT *S. gordonii* and *spxB* mutant exposures. Since PGP-5 is a membrane-associated ABC transporter protein implicated in xenobiotic resistance and cellular stress adaptation, we hypothesized that loss of PGP-5 could alter the host response to pathogen-induced stress specifically related to bacterial-derived H_2_O_2_. Therefore, we investigated whether *pgp-5* knockdown affects *zip-2*-dependent *irg-1p*::GFP expression upon *S. gordonii* exposure. Consistent with this idea, *pgp-5* knockdown resulted in a significant increase in *irg-1p*::GFP expression levels compared with empty vector control worms upon exposure to WT *S. gordonii* (Figure 5A,B). In contrast, the *S. gordonii spxB* mutant did not induce significant *irg-1p::*GFP expression under either control or *pgp-5* knockdown conditions. These results suggest that the enhanced *zip-2*-dependent *irg-1p*::GFP response observed during WT *S. gordonii* exposure is associated with *spxB*-dependent H_2_O_2_ production and altered stress tolerance following *pgp-5* knockdown. ZIP-2 knockdown served as a positive control in this experiment. As expected, *zip-2* knockdown completely abolished *irg-1p::*GFP induction following *S. gordonii* exposure, confirming that reporter activation is dependent on ZIP-2. Consistent with this finding, double knockdown of *pgp-5* and *zip-2* further confirmed that *irg-1p*::GFP activation under both OP50 and *S. gordonii*-exposed conditions is ZIP-2-dependent.

### 3.6. Validation of Microscopic Findings Using qRT-PCR

**Figure 6:**
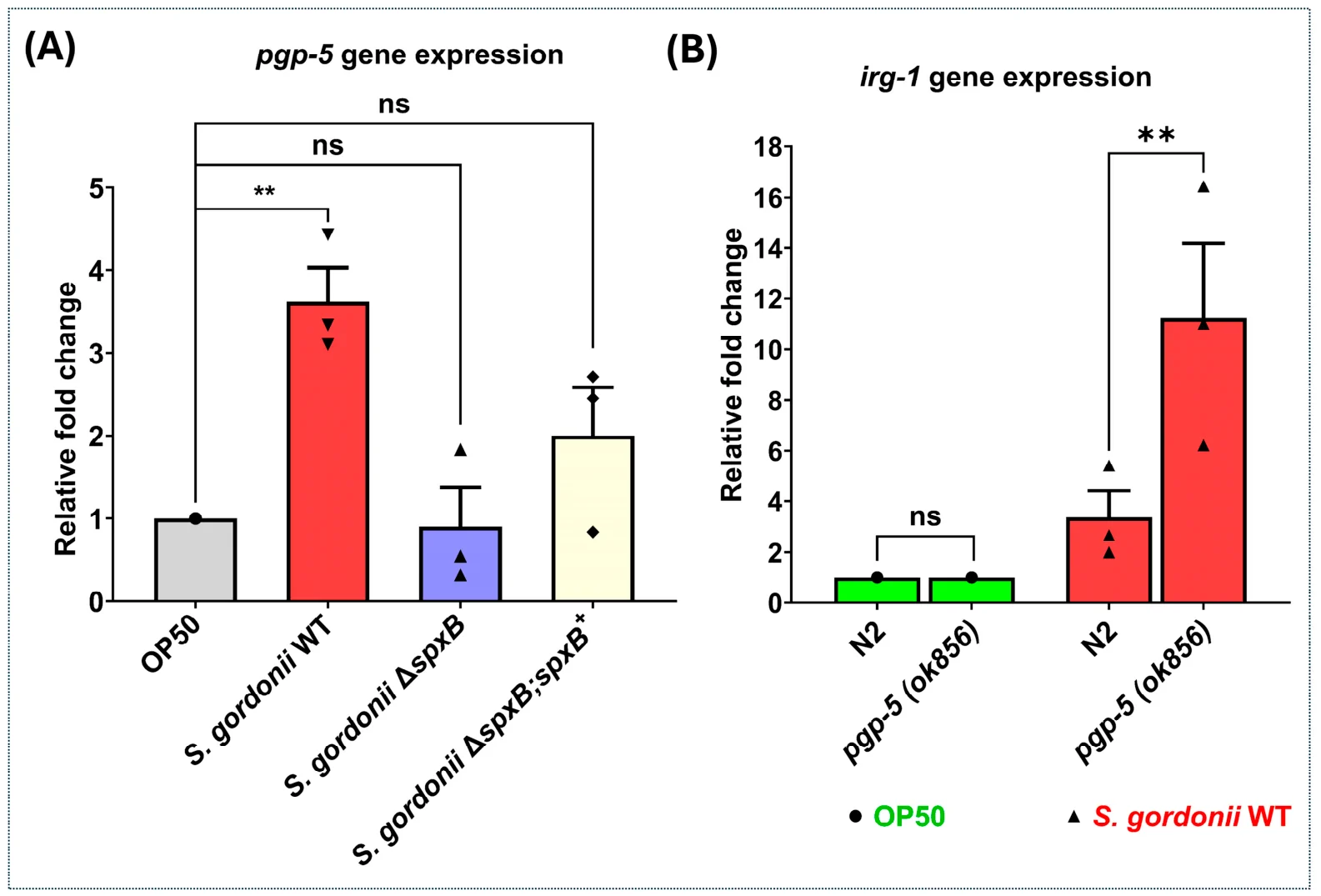
The qRT-PCR results represented here include N2 and *pgp-5*(*ok856*) mutant backgrounds. *pgp-5* and *irg-1* levels were normalized to *act-1* and compared with the OP50 condition as a baseline. (**A**) qRT-PCR analysis validates the fluorescence reporter results shown in Figure 2A, demonstrating H_2_O_2_-dependent induction of *pgp-5*. Statistical analysis was performed on ΔCt values using unpaired two-tailed Welch’s *t*-tests, with each *S. gordonii* condition compared with the OP50 control (*n* = 3). ** *p* < 0.01; ns—not significant. (**B**) qRT-PCR analysis shows a significant increase in *irg-1* transcript levels in the *pgp-5*(*ok856*) mutant during *S. gordonii* WT exposure compared with the N2 control, confirming the fluorescence microscopy findings shown in Figure 5A. Statistical analysis was performed on ΔCt values using an unpaired two-tailed Welch’s *t*-test comparing N2 and *pgp-5*(*ok856*) worms following OP50 and *S. gordonii* exposures (*n* = 3). ** *p* < 0.01; ns—not significant.

We utilized qRT-PCR analysis to further confirm the expression changes observed in the microscopic assays. The expression levels of *pgp-5* and *irg-1* were examined in N2 and *pgp-5*(*ok856*) mutant backgrounds following exposure to *S. gordonii*. In the N2 worms, WT *S. gordonii* exposure resulted in significant induction of both *irg-1* and *pgp-5* compared with OP50 controls (Figure 6A,B). In contrast, animals exposed to the *spxB* mutant strain showed reduced expression of *pgp-5*, demonstrating that *spxB* contributes to activation of the *pgp-5*-mediated host response and supporting the microscopic findings shown in Figure 2A. We next examined the response in the *pgp-5(ok856)* mutant background and observed increased *irg-1* expression following WT *S. gordonii* exposure compared with N2 worms (Figure 6B). This finding corroborates the increased *irg-1p::*GFP fluorescence observed in our reporter microscopic analyses shown in Figure 5A and further supports the conclusion that loss of PGP-5 enhances *zip-2-*dependent *irg-1* immune activation during *S. gordonii* exposure.

## 4. Discussion

**Figure 7:**
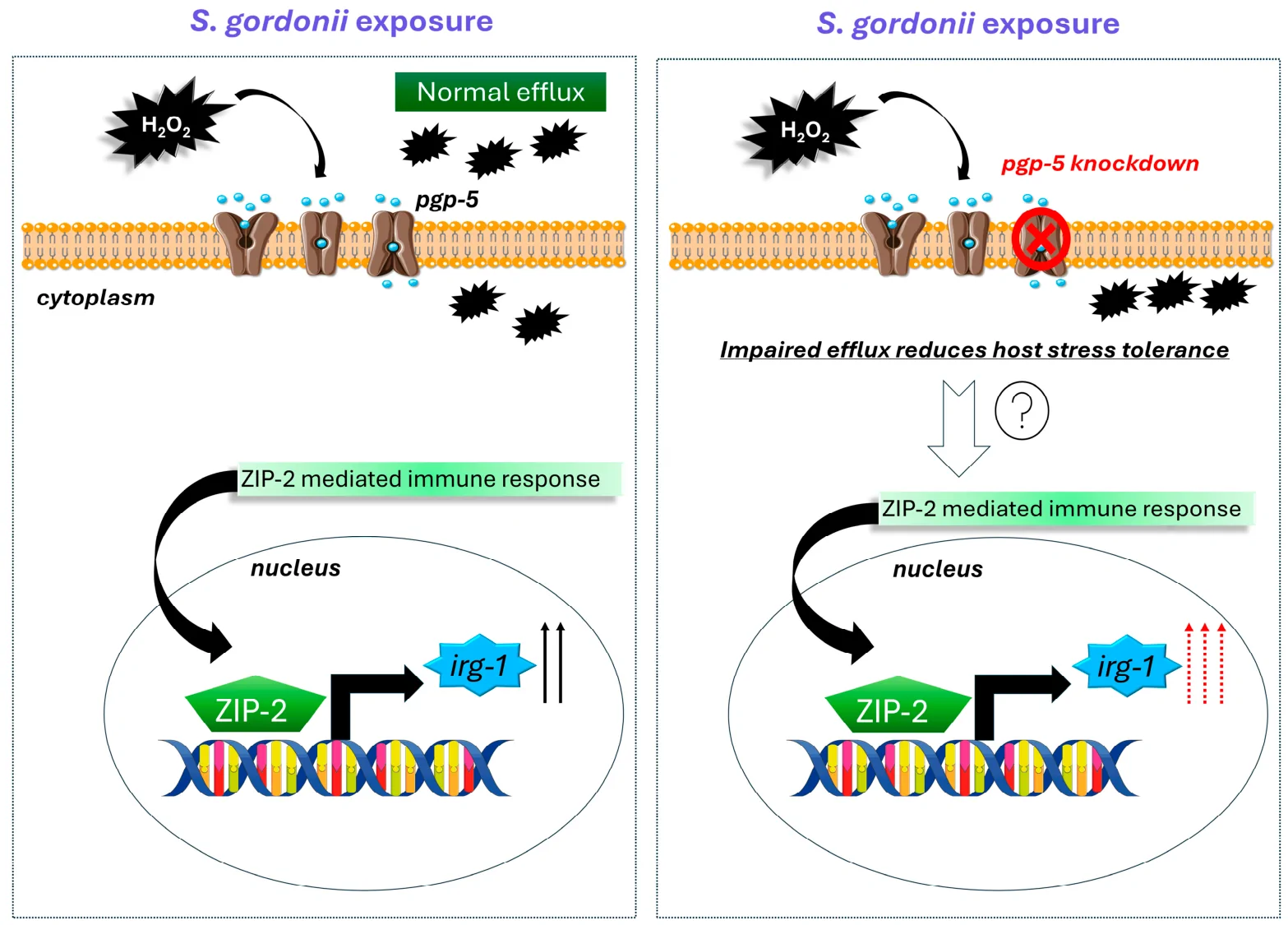
Under normal conditions, PGP-5-mediated efflux activity may contribute to cellular stress tolerance by limiting the accumulation of pathogen-derived toxic compounds. During *S. gordonii* exposure to wild-type worms, H_2_O_2_-induced stress activates the ZIP-2-dependent immune response, leading to induction of *irg-1*. Loss of *pgp-5* may reduce efflux activity and increase intracellular stress burden, which could enhance ZIP-2-responsive gene expression and lead to elevated *irg-1* expression compared with control worms. However, the mechanistic connection between PGP-5 function and ZIP-2-dependent *irg-1* induction remains to be determined. Red dotted arrows indicate enhanced gene expression and represent hypothetical relationships between PGP-5 and ZIP-2 that have not been directly demonstrated in this study. Icons used in this figure were adapted from Servier Medical Art (Servier) (https://smart.servier.com, accessed on 25 May 2026), including the ion channel-membrane and DNA-nucleotides-ribbon icons, and are licensed under CC BY 4.0 (https://creativecommons.org/licenses/by/4.0/, accessed on 25 May 2026).

Among the non-canonical innate immune surveillance pathways in *C. elegans*, ZIP-2 functions as an important regulator of host responses against pathogen-induced cellular stress [25]. Unlike the classical p38-MAPK immune pathway, ZIP-2 responds to disruptions in cellular homeostasis, particularly those associated with microbial interference of host translation. Previous studies using *P. aeruginosa* infection models and translational inhibitors, including hygromycin and Exotoxin A, identified ZIP-2 as a central component of pathogen surveillance immunity [21,25,26,33]. Upon activation, ZIP-2 regulates the expression of several infection response genes, including *irg-1*, *irg-2*, *irg-3*, etc. [25,26,34]. However, the contribution of ZIP-2 during bacterial pathogenesis with Gram-positive pathogens remains largely unexplored. In this study, we demonstrated that *spxB-*dependent H_2_O_2_ produced by *S. gordonii* activates the ZIP-2-dependent immune gene *irg-1* and promotes *pgp-5* expression. Furthermore, our results show that knockdown of *pgp-5* enhances ZIP-2-dependent *irg-1p::*GFP activation during *S. gordonii* exposure. These findings suggest that PGP-5-mediated stress tolerance influences the strength of ZIP-2-dependent *irg-1* immune activation during bacterial exposure (Figure 7). We hypothesize that PGP-5 may not directly regulate ZIP-2 signaling but instead functions to alleviate cellular stress caused by pathogen-derived toxic compounds, thereby indirectly influencing the magnitude of ZIP-2-dependent immune activation. When PGP-5 function is compromised, the resulting increase in intracellular stress may promote ZIP-2 immune activation and consequently enhance the expression of downstream immune response genes including *irg-1*. Collectively, our results reveal an important link between xenobiotic stress tolerance, pathogen-induced oxidative stress, and innate immune regulation in *C. elegans*, highlighting a previously unrecognized role for PGP-5 in modulating ZIP-2-dependent immune responses. Even though this study identifies a functional relationship between PGP-5 and ZIP-2-dependent *irg-1* response, the molecular mechanism connecting these pathways remains unclear. Whether PGP-5 affects ZIP-2 protein stability, activation, or nuclear localization requires further investigation. In addition, the upstream mechanisms controlling ZIP-2 activation, including potential regulators of its stability, post-translational modifications, and nuclear translocation, are not well defined in *C. elegans*. Future studies examining endogenous ZIP-2 nuclear localization, identifying upstream regulators, and analyzing additional ZIP-2-dependent genes such as *irg-2* and *irg-3* will help define the broader role of this immune axis under *pgp-5* knockdown and pathogenic stress conditions.

It will also be important to determine whether other P-glycoprotein family members can compensate for the loss of *pgp-5* or whether PGP-5 plays a unique function during bacterial pathogenesis. In *C. elegans*, approximately 60 ABC transporter genes have been predicted, including 14 annotated pgp genes belonging to the P-glycoprotein (PGP/ABCB) family [35]. However, only a few of these PGP transporters have been functionally characterized during stress responses and innate immunity [13,15,16,17,20,22,23,24,35,36]. Among the PGP family members, PGP-5 represents the best-characterized immune-responsive transporter; however, several other PGP proteins and closely related paralogs remain poorly understood. Further investigation of these proteins under diverse stress conditions, including pathogen-exposures may reveal additional roles beyond their established functions in xenobiotic stress tolerance and detoxification. Nevertheless, omics approaches such as interactome analysis and glycoproteomic profiling of PGP-5 may provide additional insights into how this transporter protein contributes to host defense [37,38,39,40]. Identification of PGP-5-associated proteins through interactome analysis could reveal regulatory networks that connect transporter activity with pathogen-induced stress signaling and ZIP-2 activation. In addition, because PGP-5 is predicted to be a glycosylated membrane protein, glycoproteomic analyses may reveal whether pathogen-induced changes in post-translational modification affect its stability, localization, or transport activity. Such studies would advance our understanding of how PGP-5 integrates detoxification and stress tolerance processes with innate immune signaling during bacterial pathogenesis.

From a cross-species perspective, although ZIP-2 does not have a direct mammalian ortholog, PGP-5 shares functional similarity with human ATP-binding cassette transporters, including ABCB1 and ABCB4 [41]. For instance, ABCB1 in human being functions as a broad-spectrum efflux transporter involved in xenobiotic transport, multidrug resistance, epithelial barrier protection, and inflammatory disease [42,43]. Therefore, understanding the role of PGP-5 in *C. elegans* may provide broader insights into how efflux systems influence host responses to microbial stress and toxic metabolites. In addition, our findings present a foundation for further studies aimed at understanding the contribution of this protein to host–pathogen interactions. Altogether, this study establishes *C. elegans* as a useful model for dissecting how *spxB*-mediated H_2_O_2_ produced by mitis-group streptococci influences host immunity and reveals an important connection between detoxification pathways and pathogen-responsive immune surveillance.

## 5. Conclusions

This study reveals an important connection between PGP-5-mediated stress response and ZIP-2-dependent *irg-1* expression during *S. gordonii* exposure in *C. elegans*. Our findings demonstrate that WT *S. gordonii* induces *pgp-5* and the ZIP-2-dependent immune gene *irg-1* in a *spxB*-dependent manner, while loss of *pgp-5* enhances *irg-1* expression and reduces host survival following bacterial exposure. Genetic loss of *pgp-5* resulted in an increased *irg-1* expression and reduced host stress tolerance, suggesting that PGP-5 contributes to host protection during bacterial exposure and may influence the magnitude of the pathogen-induced immune response. These findings highlight that host defense is not mediated solely by immune signaling pathways but also depends on cellular detoxification/stress tolerance mechanisms that influence the intensity of immune activation. In summary, this study expands our understanding of how efflux-mediated stress tolerance influences pathogen-induced immunity. It also provides new insights into the complex relationship between cellular homeostasis and host defense during bacterial pathogenesis. Importantly, this study provides a foundation for further investigation of how PGP-5 and ZIP-2 pathways intersect during bacterial pathogenesis.

## Figures and Tables

**Table 1:** Primers used in this study for qRT-PCR.

Gene Name	Forward (5′–3′)	Reverse (5′–3′)
*act-1*	ACCATGTACCCAGGAATTGC	TGGAAGGTGGAGAGGGAAG
*pgp-5*	TCAAGCTGTGGAGGAGGATTC	TCCATGTTAAGTGCTCGAACAG
*irg-1*	TGATCTTGTTCCGTACCCATG	TTCTCCAAGCCAGTTCGATC

## Data Availability

The raw data supporting the conclusions of this article will be made available by the authors on request.

## Abbreviations

The following abbreviations are used in this manuscript:

*C. elegans*	*Caenorhabditis elegans*
GFP	Green Fluorescent Protein
H_2_O_2_	Hydrogen peroxide
IPTG	Isopropyl β-D-1-thiogalactopyranoside
*irg-1*	Infection Response Gene
LB medium	Luria–Bertani Medium
p38-MAPK	p38-mitogen-activated protein kinase
PGP-5	P-GlycoProtein related
*P. aeruginosa*	*Pseudomonas aeruginosa*
qRT-PCR	Quantitative Reverse Transcription-Polymerase Chain Reaction
RNAi	RNA interference
*S. gordonii*	*Streptococcus gordonii*
*spxB*	Pyruvate oxidase
THY medium	Todd–Hewitt broth supplemented with Yeast extract
WT	Wild type
ZIP-2	bZIP transcription factor family
